# Primary Carcinoid Tumor of the Ovary: A Case Report With Radiologic and Pathologic Correlation

**DOI:** 10.7759/cureus.58494

**Published:** 2024-04-17

**Authors:** Dana Amiraian, Neema Patel, J. Kenneth Schoolmeester, Candice Bolan

**Affiliations:** 1 Department of Radiology, Mayo Clinic, Jacksonville, USA; 2 Department of Laboratory Medicine and Pathology, Mayo Clinic, Jacksonville, USA

**Keywords:** pathology, magnetic resonance imaging, tumor, primary, carcinoid, ovarian

## Abstract

Ovarian carcinoid tumors are very rare entities that often mimic other ovarian neoplasms. A case of primary ovarian carcinoid in a 44-year-old woman is presented with emphasis on the magnetic resonance imaging (MRI) features of the tumor and pathologic correlation. Ovarian carcinoid tumors can be variable in their MRI appearance, presumably due to different tumor subtypes and tumor components, thus requiring pathologic diagnosis. It is imperative to accurately diagnose primary ovarian carcinoid tumors, as their prognosis is usually more favorable compared to other malignant ovarian neoplasms.

## Introduction

Primary ovarian carcinoid is a rare entity with limited current literature, particularly as it relates to imaging findings. Varying imaging appearance of ovarian carcinoid is likely related to different carcinoid subtypes and tumor elements, necessitating pathologic diagnosis.

Primary ovarian carcinoid is nearly always unilateral [1,2], whereas metastatic carcinoid is typically bilateral with accompanying peritoneal disease [2,3].

Primary ovarian carcinoid generally has a better prognosis compared to poorly differentiated neuroendocrine ovarian tumors, metastatic ovarian carcinoid, and other more aggressive ovarian malignancies [2,4]. It is therefore essential to correctly diagnose primary ovarian carcinoid to facilitate timely and appropriate management.

The current case of primary ovarian carcinoid in a 44-year-old woman highlights the magnetic resonance imaging (MRI) features of the tumor with pathologic correlation, adding to the limited literature on this topic.

This article was previously presented as a poster at the 2016 Florida Radiological Society Annual Meeting on August 6, 2016.

## Case presentation

A 44-year-old gravida 2 para 2 African American woman presented with a one-year history of menorrhagia and constipation. Past medical history was significant for prior left salpingo-oophorectomy for benign mature teratoma of the left ovary. Clinical pelvic examination revealed a large, palpable, firm, non-mobile midline mass in the cul-de-sac, which was initially suspected to represent a uterine leiomyoma. Laboratory studies identified an elevated creatinine of 1.4 mg/dL. 

**Figure 1:**
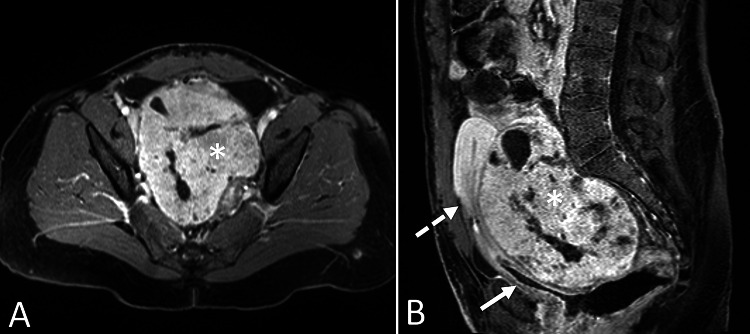
Post-contrast pelvic MRI. T1 weighted post-contrast pelvic MRI in axial (A) and sagittal (B) planes demonstrates a large, predominantly solid, and avidly enhancing mass (asterisks) occupying most of the pelvis. There is associated mass effect on the bladder (solid arrow) and uterus (dashed arrow).

**Figure 2:**
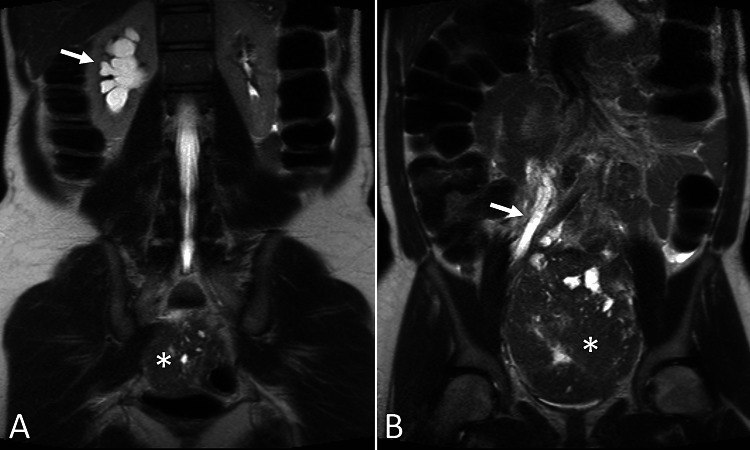
T2 weighted pelvic MRI. Coronal T2 weighted pelvic MRI demonstrates the pelvic mass with cystic components (asterisks). There is right-sided hydronephrosis (arrow in A) and hydroureter (arrow in B) due to distal ureteral obstruction by the mass.

**Figure 3:**
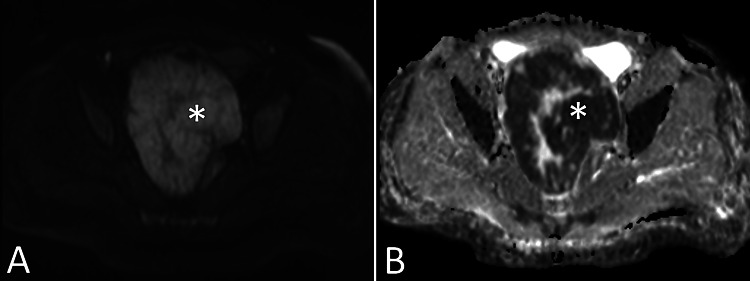
Diffusion weighted pelvic MRI. Axial diffusion weighted imaging (A) and ADC map (B) from the pelvic MRI demonstrate diffusion restriction of the solid portions of the pelvic mass (asterisks), with hyperintensity on diffusion weighted imaging and corresponding hypointensity on ADC. ADC: Apparent diffusion coefficient

Pelvic MRI was performed for further evaluation and revealed a 10.5 x 14.0 x 13.0 cm circumscribed, predominantly solid mass with cystic components occupying most of the pelvis (Figures 1, 2). Solid portions of the mass demonstrated avid contrast enhancement (Figure 1) with restricted diffusion (Figure 3). There was significant mass effect on the bladder, uterus, and right ureter, with associated severe right hydroureteronephrosis (Figures 1, 2). No ascites or peritoneal implants were identified.

**Figure 4:**
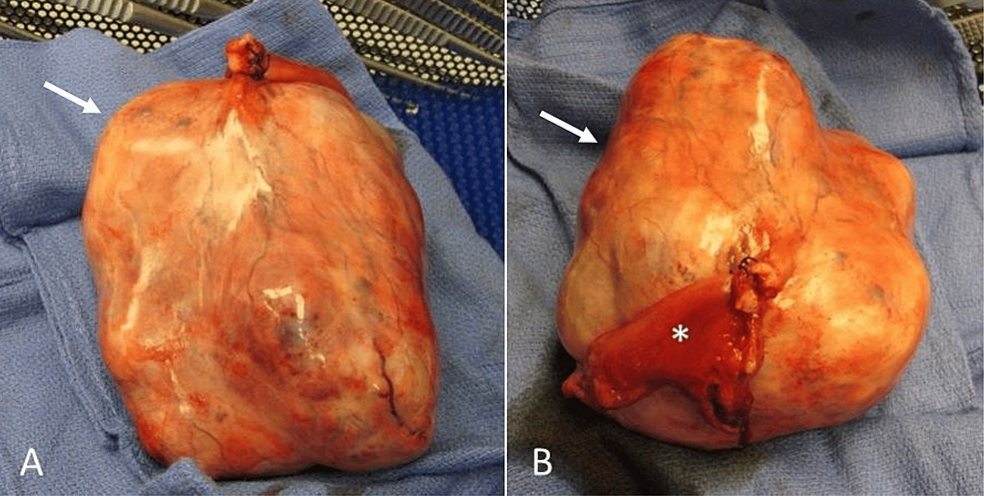
Surgical pathologic specimen. A surgical pathologic specimen of ovarian carcinoid obtained from right salpingo-oophorectomy demonstrates a large, firm, solid, circumscribed, tan-yellow right ovarian mass (arrows), measuring about 14 cm in greatest dimension. The right fallopian tube is also seen (asterisk in B).

**Figure 5:**
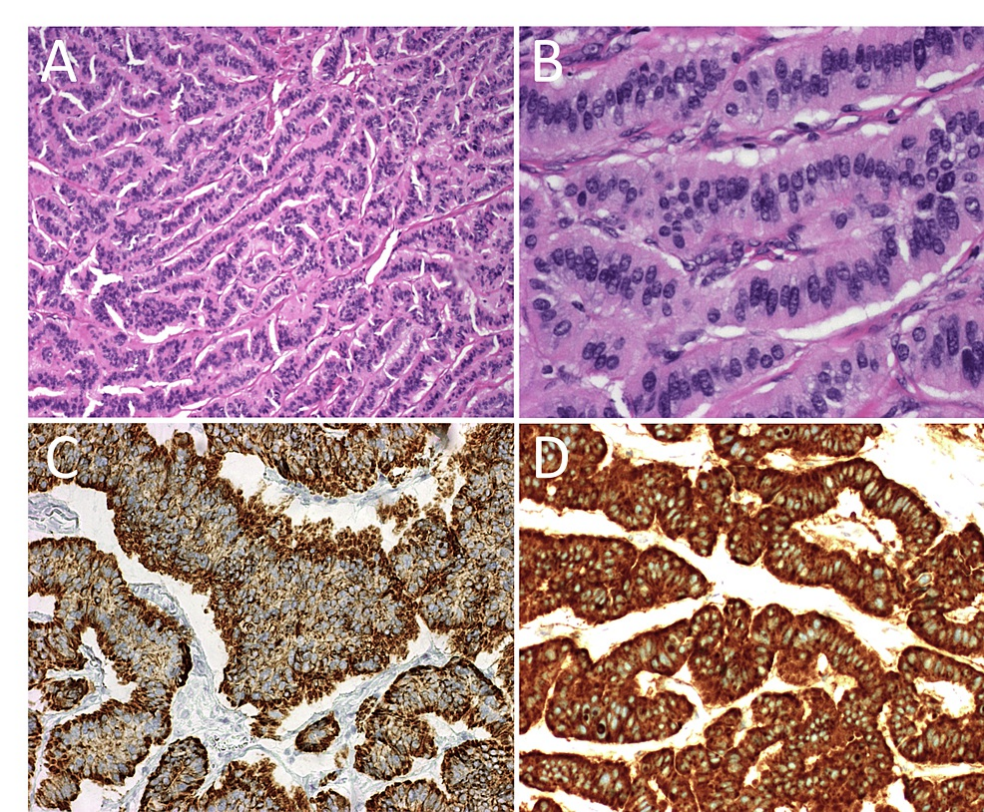
Histopathology. Histopathologic images of the ovarian trabecular carcinoid show thin ribbons and cords of tumor (A) composed of elongated to columnar cells with eosinophilic cytoplasm and nuclei containing fine ‘salt and pepper’ chromatin (B). Immunohistochemistry demonstrates expected diffuse and strong expression of broad-spectrum keratins (C) and chromogranin A (D).

The patient subsequently underwent exploratory laparotomy, right salpingo-oophorectomy, hysterectomy, right para-aortic lymph node sampling, partial infracolic omentectomy, cystoscopy with right ureteral stenting, and peritoneal washings. A gross pathologic specimen obtained at surgical resection demonstrated a large, firm, solid, tan-yellow right ovarian mass (Figure 4). Final histopathology (Figure 5) demonstrated a trabecular growth pattern lined by uniform appearing neoplastic cells containing monotonous nuclei with a salt and pepper chromatin appearance, typical of carcinoid tumors. Immunostaining was positive for synaptophysin and chromogranin, consistent with carcinoid tumors. There was no evidence of metastasis.

Following surgical resection, imaging with computed tomography (CT) of the chest, abdomen, and pelvis confirmed no evidence of residual, recurrent, or metastatic disease. Eight years after resection, the patient has no known evidence of disease.

## Discussion

Ovarian carcinoid is quite rare, comprising less than 1% of all carcinoid tumors and less than 1% of all malignant ovarian tumors [1,2,4-6]. Ovarian carcinoid may be primary or metastatic. Primary ovarian carcinoid is usually unilateral [1,2], while metastatic carcinoid is usually bilateral with associated peritoneal disease [2,3].

Primary ovarian carcinoids are monodermal teratomas and can be divided into four pathological subtypes: insular, trabecular, mucinous, and strumal [1,4,7,8]. Insular carcinoid is the most common type [8]. Insular subtypes are malignant but often demonstrate slow growth with a low likelihood of metastatic disease [9]. Mucinous carcinoids are more aggressive and may present with metastases [9]. Trabecular and strumal subtypes are not typically associated with metastatic disease [9].

Ovarian carcinoid typically occurs in peri- or post-menopausal women [1,3,7,8,10,11]. Clinical presentation depends on the size of the tumor, as well as the amount and type of carcinoid tissue [5,8]. Many ovarian carcinoid tumors are asymptomatic [1], though abdominal pain or features of mass effect can be present [2,8]. Up to approximately one-third of patients with insular type carcinoid may present with carcinoid syndrome with classical flushing and diarrhea [1,2,8,11]. Carcinoid syndrome can be present in the absence of hepatic metastases due to systemic drainage via the gonadal (ovarian) veins, bypassing the portal system [2,5,7,8].

There is limited literature on the imaging findings of ovarian carcinoid tumors. This is likely due to the rarity of primary ovarian carcinoid, as well as the variable tumor types and contents. Ovarian carcinoid can arise in association with a mature cystic teratoma [2,4,8,10], with imaging features of cystic teratoma with a solid component. However, these lesions can also be seen as an isolated mass [2-4,8], in which case the imaging more often follows that of a solid ovarian mass, with a solid mass differential. In association with a teratoma or in isolation, the solid component has been described as hypointense on T2 weighted MRI, which is thought to be related to dense fibrous stroma [1]. Solid tumor components are also described as demonstrating contrast enhancement and diffusion restriction, suggesting hypervascularity and hypercellularity, respectively [1]. Mucinous ovarian carcinoid is noted to be relatively hyperintense on T2 weighted MRI due to higher mucin content [10,12]. Overall, the imaging findings can be nonspecific, and imaging often cannot differentiate ovarian carcinoid from other ovarian neoplasms.

Pathologic evaluation is therefore essential in diagnosing ovarian carcinoid tumors. Staining for neuroendocrine markers such as synaptophysin and chromogranin helps confirm the diagnosis [4,7].

The majority of primary ovarian carcinoid tumors follow a benign course [2,4], which offers a much more favorable prognosis compared to other more aggressive ovarian malignancies. On the other hand, poorly differentiated neuroendocrine tumors of the ovary may present with metastatic or advanced disease and therefore portend a worse prognosis [4].

Surgical resection with negative margins is the mainstay of treatment [3,4]. This is almost always curative in early-stage disease [3,6]. However, poorly differentiated neuroendocrine tumors and metastatic ovarian carcinoid may require additional management, such as radiation and/or chemotherapy [4]. 

## Conclusions

Ovarian carcinoid is a rare but important diagnosis to consider in patients with an ovarian neoplasm. Imaging characteristics of ovarian carcinoid are variable and nonspecific, so pathologic diagnosis is required. The prognosis for primary ovarian carcinoid is often much more favorable compared to other ovarian malignancies due to the generally indolent course of this tumor. Therefore, timely and accurate diagnosis of ovarian carcinoid is essential for appropriate patient management.

## References

[1] Takeuchi M, Matsuzaki K, Uehara H (2011). Primary carcinoid tumor of the ovary: MR imaging characteristics with pathologic correlation. Magn Reson Med Sci.

[2] Robboy SJ, Norris HJ, Scully RE (1975). Insular carcinoid primary in the ovary: a clinicopathologic analysis of 48 cases. Cancer.

[3] Somak R, Shramana M, Vijay S, Nita K (2008). Primary carcinoid tumor of the ovary: a case report. Arch Gynecol Obstet.

[4] Vora M, Lacour RA, Black DR, Turbat-Herrera EA, Gu X (2016). Neuroendocrine tumors in the ovary: histogenesis, pathologic differentiation, and clinical presentation. Arch Gynecol Obstet.

[5] Shanbhogue AK, Shanbhogue DK, Prasad SR, Surabhi VR, Fasih N, Menias CO (2010). Clinical syndromes associated with ovarian neoplasms: a comprehensive review. Radiographics.

[6] Athavale RD, Davies-Humphreys JD, Cruickshank DJ (2004). Primary carcinoid tumours of the ovary. J Obstet Gynaecol.

[7] Eichhorn JH, Young RH (2001). Neuroendocrine tumors of the genital tract. Am J Clin Pathol.

[8] Talerman A (1984). Carcinoid tumors of the ovary. J Cancer Res Clin Oncol.

[9] Shaaban AM, Rezvani M, Elsayes KM (2014). Ovarian malignant germ cell tumors: cellular classification and clinical and imaging features. Radiographics.

[10] Choudhary S, Fasih N, Mc Innes M, Marginean C (2009). Imaging of ovarian teratomas: appearances and complications. J Med Imaging Radiat Oncol.

[11] Scully RE (1979). Tumors of the ovary and maldeveloped gonads. Atlas of Tumor Pathology.

[12] Outwater EK, Siegelman ES, Hunt JL (2001). Ovarian teratomas: tumor types and imaging characteristics. Radiographics.

